# The "Pall Mall Gazette" and the Hospitals

**Published:** 1892-01-02

**Authors:** 


### THE "PALL MALL GAZETTE" AND THE HOSPITALS.

In our last number we published a letter addressed to the *Pall Mall Gazette*, which appeared in that paper on the 23rd ult., with the following note :—

Mr. Burdett protests too much—innocence. He may not have read the criticisms on the London Hospital administration in the *Pall Mall Gazette*, nor written the eulogies on the administration in THE HOSPITAL ; but we have fully explained in the former our charge of "obscurantism," and the latter has made itself conspicuous among the specialist press devoted to these subjects by its thick-and-thin defence of the existing state of things. As for the newspaper heading "Hospital Scandal," or "More Hospital Scandals," it is familiar to every newspaper reader in connexion with the London Hospital (three sensational incidents during this year as well as the general nursing controversy), the "Eastern Hospital Scandals" (see London press for months), the *Truth* campaign against the St. Bartholomew's sanitation, the recent Glasgow Infirmary scandals (see Glasgow press, &c., &c., &c.)—ED. *P.M.G.*

The following letter was sent in reply, but was not published :—

Sir,—In your article of the 18th inst. you made no distinction, but condemned by implication all hospitals and all hospital officials, at any rate in London, as the subject-matter of the article referred to London only, "for their blind and fatal obscurantism." You now state that your allusions are based upon the criticisms which have been published in relation to four hospitals, and four only, viz., St. Bartholomew's, the Eastern Hospital, the Infirmary, Glasgow, and the London Hospital. The Glasgow Infirmary is put out of court by the terms of your leader, and is not admissible by your own showing. St. Bartholomew's Hospital is a great endowed institution living upon the revenues it derives from invested property, and does not ask for or receive aid from the public. It is, in fact, an endowed and not a voluntary hospital. The Eastern Hospital is supported entirely from the rates, and is under the control of the ratepayers.

Hence the only evidence you have left in support of your original statement is "the criticism on the London Hospital." I wish first to point out that most, if not all, of this criticism occurred before the publication of "In Darkest England," and cannot, therefore, be quoted in support of your original statement. It is true, though little to the point, that a very few persons have deliberately set themselves to do the London Hospital all the harm they can by attending recent Courts of Governors and repeating charges which have been answered, or shown to be ill-founded over and over again. The "three sensational incidents during this year" to which you refer I should like to have in detail, as they are new to me.

In these circumstances, are you still prepared to contend that your original statement is warranted when the evidence on which it is based is found to rest upon an allusion to "three sensational incidents" at one hospital, of which neither particulars nor evidence are forthcoming?

You accuse me of innocence. Might I not justly apply a less complimentary epithet in criticism of your action in this matter?—Yours faithfully, HENRY C. BURDETT.

Mr. Burdett and THE HOSPITAL were again attacked in a leading article which appeared in the *Pall Mall Gazette* of the 26th ult. In this article the Glasgow Infirmary and the Eastern Fever Hospital were quoted as instances in support of a charge that the hospital authorities wring their hands, and are guilty of mean endeavours to play upon the better feelings of the energetic and devoted class of women who constitute the nurses. We shall deal with the nursing part of the controversy in the "Mirror" next week, but meanwhile we think it well to publish the following further letter, which was addressed to the *Pall Mall Gazette* on the 26th inst., and published in their issue of the 29th, with the appended note :—

Sir,—Although you have not published my reply to your editorial note to my letter in the *Pall Mall Gazette* of 23rd inst., I must ask you, in common fairness, to give me a brief space to deal with this and the attack made in your leading article of Saturday. In the editorial note you quote four hospitals in justification of your charges against the great voluntary hospitals, viz. : St. Bartholomew's, the Eastern Hospital, the Glasgow Infirmary, and the London. The first three institutions have nothing whatever to do with your original charge, as the first is an endowed hospital, which does not receive one penny from the public ; the second is a rate-supported hospital, and the third is a Scotch hospital, whereas you were dealing only with London and London institutions. So far as the London Hospital is concerned, the criticisms to which you refer were almost entirely made before the issue of the "In Darkest England" appeal. But granting, for the sake of argument, that the London Hospital is a case in point—although I am certain the evidence is mainly the other way—there are about sixty voluntary hospitals in the metropolis, and is it fair to condemn all for the sins of one?

I pass over your remarks personal to myself, because I must stand or fall by my work for the hospitals and the nurses, and that I am content to be judged by. Is it not a burlesque of language, however, to characterise a homily in favour of kindly co-operation and friendly feeling amongst all classes of hospital workers as "canting twaddle?"

I desire to offer you a fair challenge, which I hope, in the public interest, you will see your way to accept. If you will formulate, in any form you may prefer, "The Hospital Scandals," and any instances where the hospital authorities "have been fairly 'cornered,'" and if you will give me an equal amount of space to reply, I will undertake to show, so far as the voluntary hospitals of London are concerned, that the charges are either too trivial to be, of any real importance, or that they are altogether unfounded.

I very heartily thank you for your response to my appeal, so far as you have brought out the urgent need there is at the present time for further funds for the hospitals. In my evidence before the Lords' Committee I specially dwelt upon the importance of strengthening hospital committees by the introduction of new and younger men, and I hope your appeal to the public to give more money, and more of themselves in the way of personal service to the hospitals, will be generally responded to with alacrity.—I am, Sir, your obedient servant, HENRY C. BURDETT.

[We have never "condemned all (the hospitals) for the sins of one." The words which we used about the hospitals in general are on record in our issue of Friday, December 18th. They are perfectly clear and simple, and to them we must refer those readers (if any) who care to pursue the verbal issue which Mr. Burdett raises. The "homily" of Mr. Burdett, which we have described as "canting twaddle," is also quoted in our issue of Saturday, December 26th, and of that also readers can form their own judgment. The general question of hospital administration we have discussed at length on proper occasions.—ED. *P. M. G.*]

The correspondence has thus ended by the *Pall Mall Gazette* declining to join issue on the facts and giving up the case it attempted to make against the hospitals. To make this perfectly clear it is only necessary to quote the *ipsissima verba* used in the articles referred to by the Editor of the *Pall Mall Gazette* in the above note. They are as follows :—

DECEMBER 18TH.—Extract from *Pall Mall Gazette*.—"The London hospitals spend every year six times the capital sum which the General asked for. . . . It is the year in which 'More Hospital Scandals' has become a familiar heading in the press, and which has shown us a blind and fatal obscurantism on the part of hospital officials" (*i.e.*, all London hospital officials).

DECEMBER 26TH.—Extract from THE HOSPITAL described by the *Pall Mall Gazette* as "canting twaddle":—"It is not our purpose to be severe at all, but only to express the very emphatic conviction that the strife which has been excited in various quarters is seriously to be deplored, and that there never can be either prosperity or happiness at any hospital unless the lay managers, the doctors, and the nurses live in unity of spirit and in the bond of peace. They must cease their foolish bickerings. The wiser among them must lead the less wise in the direction of peace. The nurse has been the cause of their quarrels; the nurse must unite them again. Is not this one of the noblest of the duties to which man or woman can be called—to make peace where before were rage and war?"

We agree with the *Pall Mall Gazette* that "the words used are perfectly clear and simple." They can only mean (1) that all London hospitals were included in the original charge, and (2) that the homily described as "canting twaddle" is one in favour of kindly co-operation and friendly feeling among all classes of hospital workers.

